# Does DNA Exert an Active Role in Generating Cell-Sized Spheres in an Aqueous Solution with a Crowding Binary Polymer?

**DOI:** 10.3390/life5010459

**Published:** 2015-02-09

**Authors:** Kanta Tsumoto, Masafumi Arai, Naoki Nakatani, Shun N. Watanabe, Kenichi Yoshikawa

**Affiliations:** 1 Graduate School of Engineering, Mie University, Mie, 514-8507, Japan; E-Mail: tsumoto@chem.mie-u.ac.jp; 2 Faculty of Life and Medical Sciences, Doshisha University, Kyotanabe, 610-0394, Japan; E-Mails: arai@dmpl.doshisha.ac.jp (M.A.); nakatani@dmpl.doshisha.ac.jp (N.N.); watanabe@dmpl.doshisha.ac.jp (S.N.W.)

**Keywords:** DNA, dextran, polyethylene glycol (PEG), crowding, aqueous two-phase system (ATPS), optical tweezers, microcompartment, artificial cell model

## Abstract

We report the spontaneous generation of a cell-like morphology in an environment crowded with the polymers dextran and polyethylene glycol (PEG) in the presence of DNA. DNA molecules were selectively located in the interior of dextran-rich micro-droplets, when the composition of an aqueous two-phase system (ATPS) was near the critical condition of phase-segregation. The resulting micro-droplets could be controlled by the use of optical tweezers. As an example of laser manipulation, the dynamic fusion of two droplets is reported, which resembles the process of cell division in time-reverse. A hypothetical scenario for the emergence of a primitive cell with DNA is briefly discussed.

## 1. Introduction

In living cells, DNA molecules are stored inside microcompartments, such as nuclei, where RNA and proteins exist in rather high concentrations. These macromolecules, mainly RNA and protein partly with DNA, are present at concentrations of 0.3–0.5 g/mL [1,2,3]; *i.e.*, the intracellular environment is very crowded, in contrast to the usual conditions in *in vitro* biochemical experiments. Thus, it would be desirable to perform physico-chemical studies on the structure and function of DNA molecules in a crowded environment. As a simple artificial system analogous to the interior of cells composed of various kinds of polymers, an aqueous two-phase system (ATPS), which is a polymer-containing immiscible solution that has been studied for more than one hundred years, has recently received renewed attention with respect to scientific interest in micrometer-scale systems, as mentioned below [4,5,6,7]. As comprehensively reviewed by Albertsson [8], and more recently by Molino *et al.* [9], the ATPS can be used to separate almost any biological materials, such as DNAs, RNAs, proteins, virions, cells, *etc.*, due to its mild and simple protocols as well as high efficiency. In addition, improvements through the use of novel protocols have been reported [10]. Generally, two or more hydrophilic polymers that have different structures in their main chains are added to a single aqueous solution, which then becomes opaque upon slight mixing. The immiscible solution can be easily separated into two or more layered phases through simple standing or centrifugation. Since the system composed of dextran and polyethylene glycol (PEG) has been investigated for decades as a simple ATPS, detailed phase diagrams of various dextran/PEG ATPSs have been published [8]. Accordingly, one can select suitable conditions, including temperature, pH (buffers), concentrations of polymers and coexisting salts, *etc.*, to separate biological macromolecules; generally, such target macromolecules have a distinct affinity for either the dextran-rich phase, or the PEG-rich phase, or their interface under a given condition.

For conventional practical use, an ATPS is preferably prepared in a medium-sized vessel, whereas the ATPS in micrometer-sized spaces has recently become more attractive because of its distinctive ability to exhibit partitioning. A series of pioneering studies by Keating’s group [4,11,12] showed that if a dextran/PEG solution that was homogenized above the critical temperature was encapsulated by micrometer-sized lipid vesicles, then two immiscible phases could emerge in the interior as the temperature decreased. According to those reports, such an encapsulated ATPS could distribute not only inner macromolecules but also the lipid membranes of the host vesicles according to their affinities. They considered that these microcompartments in small ATPSs could be associated with intracellular organization [4,11,12]. Microscopic ATPSs could also be used as fluid materials in microfluidic devices to stabilize immiscible parallel flows in a single channel to keep DNA molecules dissolved in one of the flows [7].

To efficiently distribute target materials, in general it is better to set a dextran/PEG composition far from the critical point on the binodal curve of a phase diagram because phase segregation proceeds rapidly and completely. On the other hand, if the dextran/PEG composition is set at or near the critical point, phase segregation proceeds slowly; if the mixture is allowed to simply stand, it continues to be turbid for a long time. This indicates that aqueous solutions of the two polymers microscopically exhibit phase segregation to form microsphere-type structures, which, of course, are not stable from an equilibrium perspective. Although they occur within zones that seem to be unsuitable for the separation of materials, such micrometer-sized microspheres can sharply include/exclude biological macromolecules, just like large-scale ATPS. In this short note, we demonstrate this phenomenon through the microscopic observation of DNA molecules that were encapsulated within a dextran-rich microsphere or excluded from a PEG-rich microsphere. This entrapment was so robust that microspheres harboring DNA aggregates could be transported through the use of optical tweezers, and, in some cases, fused to each other.

## 2. Experimental Section

### 2.1. Materials

According to the report by Toyama *et al.* [13], we used dextran (200,000; for leukocyte separation) and polyethylene glycol (PEG) (6000; molecular biology grade), which we obtained from Wako Pure Chemical Industries (Osaka, Japan). DNA (from salmon sperm; for molecular biology) was purchased from Wako and dissolved in water at 0.11 mg/mL. These polymers and DNAs were dissolved in distilled pure water.

### 2.2. Preparation of the ATPS

Stock solutions of dextran and PEG were prepared by dissolving these polymers in water to give 20 (w/w) solutions. For the preparation of dextran/PEG aqueous solutions of various concentrations, we mixed the above stock solutions with water to obtain a 1-mL system. As mentioned below, if an ATPS formed, the mixture became turbid upon vortex mixing.

### 2.3. Microscopic Observation and Optical Manipulation with Laser Tweezers

An aliquot of an ATPS solution was placed between two glass spacers (0.1 mm in thickness) on a glass cover slip (30 mm × 40 mm) and covered with another cover slip. Microscopic images were obtained using a Nikon TE-300 inverted microscope equipped with a CCD camera (WAT-120N; Watec Co., Ltd., Tsuruoka, Japan). The microscope was additionally installed with an optical laser manipulation system (Millennia IR; Spectra-Physics, Tokyo, Japan; Nd:YAG, 1064 nm). Samples were prepared and observed at room temperature (near 23 °C).

## 3. Results and Discussion

**Figure 1:**
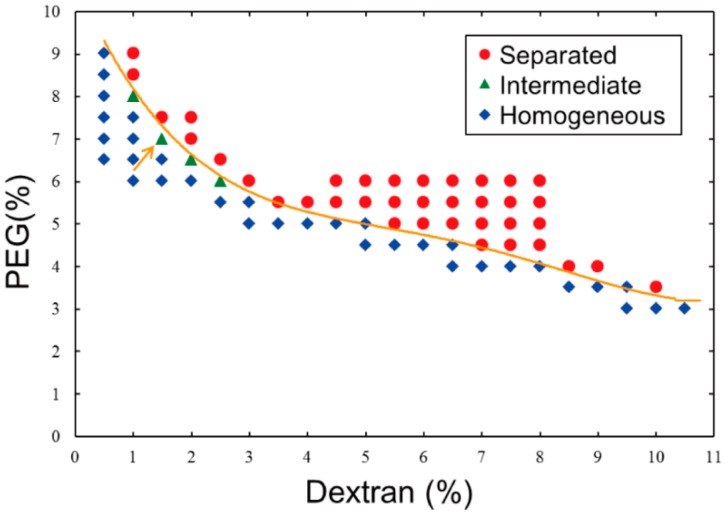
Phase diagram of an aqueous two-phase system (ATPS) composed of dextran/polyethylene glycol (PEG) at 23 °C. Data were obtained experimentally based on the phase segregation of mixtures with various concentrations of the two polymers. The behavior at each point is described as *separated* (red circle), *intermediate* (green triangle), or *homogeneous* (blue diamond). A line is drawn to show a boundary that was experimentally determined here for convenience. The condition indicated by an arrow (1.5% dextran, 7% PEG) was used in experiments shown in the subsequent figures.

**Figure 2:**
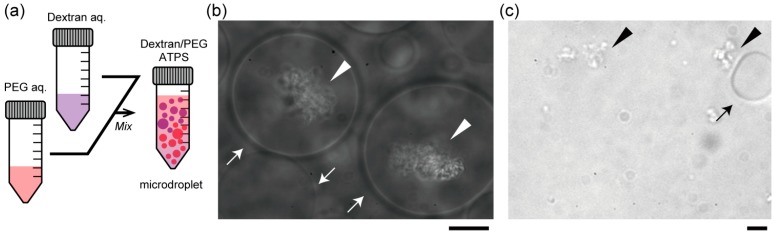
Entrapment of DNA aggregates inside dextran-rich microdroplets. (**a**) Schematic illustration of a dextran/PEG ATPS containing microspheres near a binodal curve. In the upper region, dextran-rich droplets are surrounded by PEG and in the lower region, *vice versa*. Phase contrast microscopic images show dextran-rich droplets (in the upper region) that encapsulated DNA aggregates (**b**) and PEG-rich droplets (in the lower region) from which DNA was excluded (**c**). Arrows and arrowheads indicate microdroplets and DNA aggregates, respectively. Dextran and PEG are 1.5% and 7% PEG. Bar: 10 µm.

Referring to the phase diagram of segregation in a dextran/PEG ATPS that was reported by Toyama *et al.* [13], we prepared a phase diagram (Figure 1) with our experimental scales and materials because the behavior of an ATPS near around a critical point and/or a binodal curve generally tends to fluctuate. To simply verify whether or not an ATPS at a certain point (composition) exhibits segregation, the following procedure is necessary: stock solutions of dextran and PEG are added with pure water to a micro-test tube, and the resulting solution is mixed vigorously using a vortex mixer and then either allowed to stand or briefly centrifuged. If a mixture solution remained turbid even after standing/centrifugation, the composition could be considered to be near a critical point, which means that clear separation does not occur (the compositions are indicated as “Intermediate” in Figure 1). Practically, we adopted the following criteria to determine whether or not a solution was near criticality: Under a critical condition, a turbid state might indeed last for some duration, but phase segregation could also proceed slowly. As a result, in the upper part of the tube, microdroplets containing dextran-rich solutions formed and were surrounded by PEG-rich solutions, whereas in the lower part of the tube, microdroplets containing PEG-rich solutions were observed microscopically and were surrounded by dextran-rich solutions (Figure 2a). The boundary in the diagrams, *i.e.*, an apparent binodal curve, could be shifted sensitively due to a slight change in the environment, such as in the temperature. The reason why intermediate states might not be observed with higher dextran concentrations is because a solution containing higher-concentrated dextran is apt to be segregated into two phases relatively faster than that containing lower-concentration dextran due to difference in density between dextran and PEG. Even after mixing, solutions with high dextran concentration (that is, low PEG concentration) could not be stable in an intermediate state. In other words, the compositions indicated as Intermediate in Figure 1 seem to be in intermediate states stably only for some duration. It is noted that more rapid and/or longer centrifugation on such intermediate solutions could cause segregation into two phases.

DNA molecules are generally partitioned almost completely into either dextran (bottom) or PEG (top) phase, which preference may decisively depend on the composition of the polymers and coexisting salt and the base content, length and helix structure of DNA [8]. With the condition used here, DNA molecules have apparently affinity for the dextran phase, or are excluded from the PEG phase macroscopically. Figure 2b,c show the specific localization of salmon DNA in droplets of the ATPS solution near criticality. Condensed DNA is observed with a phase contrast microscope. Figure 2b shows typical images of dextran-rich microdroplets in the presence of DNA. As expected, DNA aggregates were mostly associated with the dextran-rich phase microscopically. Specifically, in the upper part, DNA aggregates were encapsulated inside dextran-rich droplets, whereas DNA existed outside PEG-rich droplets in the lower part (Figure 2c). This suggests that DNA molecules may prefer dextran-rich regions in an ATPS even though they assume microdroplet shapes surrounded by PEG.

**Figure 3:**
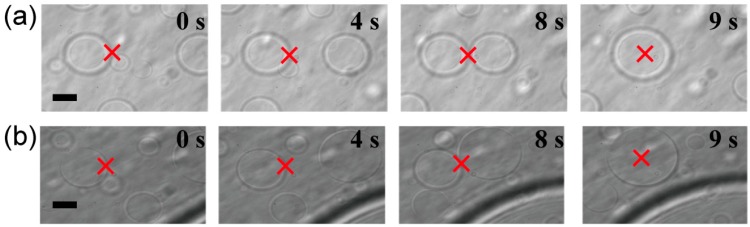
Trapping, transport and fusion of microspheres in an ATPS consisting of dextran and PEG. (**a**) The upper phase contained dextran-rich microspheres in a PEG-rich exterior solution. (**b**) The lower phase contained PEG-rich microspheres in a dextran-rich exterior solution. In both cases, microspheres were successfully fused. Times after trapping are indicated. The focus point of the laser is marked by crosses as a guide. Dextran and PEG are 1.5% and 7% PEG, respectively. Bar: 10 µm.

As has been reported for some decades, macromolecular assemblies and aggregates such as giant DNA condensates [14], giant unilamellar liposomes [15], *etc.*, can be directly trapped and manipulated using optical tweezers. In the present study, we verified whether microspheres could be trapped in the ATPS using a laser. Figure 3 shows that dextran- or PEG-rich microspheres with diameters larger than 10 µm were transferred, attached to other spheres, and then fused to become larger spheres.

**Figure 4:**
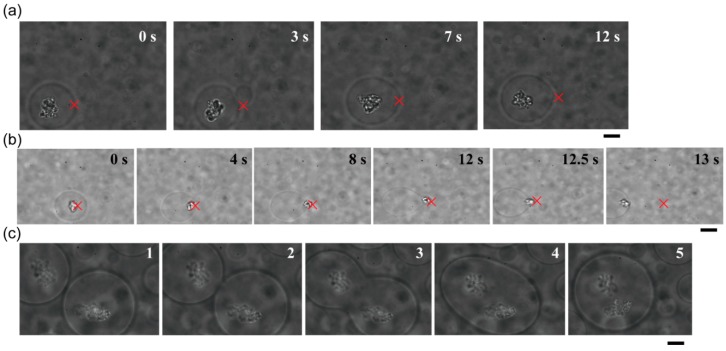
Transport and fusion of DNA-containing dextran-rich microspheres using optical tweezers. (**a**) The target microsphere could be transferred while it entrapped DNA aggregates. (**b**) A DNA aggregate trapped by a laser could not be moved across the interface between a dextran microsphere and the outer PEG solution. The focus point of the laser is marked by crosses as a guide. (**c**) Two DNA-entrapping microspheres spontaneously fused with each other. The first photograph of this series is the same as that in Figure 2b. In (a) and (b), the numbers in the upper right are the time after trapping; in (c), the numbers simply indicate the order in which images were captured. Dextran and PEG are 1.5% and 7% PEG, respectively. Bar: 10 µm.

Both microspheres and DNA aggregates can be trapped using a laser. Therefore, encapsulated DNA aggregates could be transported by applying optical tweezers to the target dextran microspheres (Figure 4a). Due to the apparently strong associations between DNA and dextran droplets, DNA aggregates that had been trapped under a laser focus could not be extracted from the microsphere (Figure 4b). Interestingly, we observed that a single DNA-trapping microsphere was trapped and moved to be juxtaposed to another DNA-trapping microsphere, leading to their spontaneous fusion into a larger droplet containing two DNA aggregates (Figure 4c).

**Figure 5:**
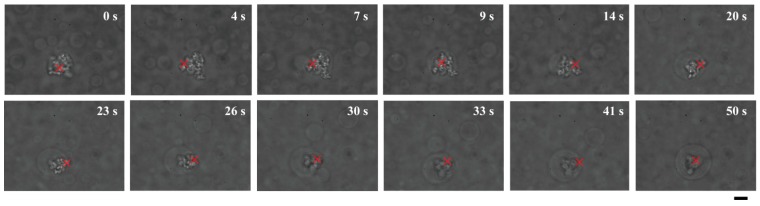
Direct observation of growth of a dextran microdroplet around a core of DNA aggregates trapped under laser radiation. Times after trapping are indicated. A growing dextran droplet became a cell-like structure containing DNAs inside at 50 s. Dextran and PEG are 1.5% and 7% PEG, respectively. Bar: 10 µm.

These results raise the question of how such DNA-entrapping dextran microdroplets emerge. Most plausibly, DNA might enter dextran microdroplets that have already formed due to the exclusionary effects of PEG solutions. Alternatively, dextran microspheres might emerge and grow larger around cores of DNA aggregates. Interestingly, dextran apparently gathered to form a spherical shape around DNA molecules that were trapped under laser radiation in PEG solutions (Figure 5). Figure 1 shows that, near a critical point/binodal curve, the behavior of such a binary polymer solution can be easily influenced by a change in the environment, such as a change in temperature. Thus, it could be implied that if heating by a laser that traps DNA aggregates induces micrometer-scale phase separation of dextran, dextran must be concentrated around the aggregates through apparent affinitive interactions.

As is well known, genomic DNA is surrounded by highly concentrated biopolymers such as proteins, RNAs, *etc.*, and DNA chains are condensed to some extent. Generally, such localization of DNA molecules in the center of nuclei/cells is regulated in a complicated manner by spatially organized genetic products. On the other hand, recent studies in actual living cells have found that some intracellular and intranuclear structures are naturally organized through liquid-liquid phase separation (LLPS) based on the accumulation of the weaker interactions of biomolecules [16,17,18]. Although the experimental systems we investigated in the present study are quite simple and definitely different from the conditions in actual cells, they exhibited cell-like morphologies. Therefore, we propose a working hypothesis that DNA is “nuclear” in nature, *i.e.*, it can serve as a core for the development of such morphology, under macromolecular crowding, probably because of its properties as a stiff polyelectrolyte. Of course, the potential of DNA molecules must be examined more comprehensively from a biological perspective, including their effects on their own genetic activities.
